# Supplementary material for: The Evolution and Characterization of the RNA Interference Pathways in Lophotrochozoa
Source: Genome Biol Evol. 2024 May 7;16(5):evae098. doi: 10.1093/gbe/evae098 (PMC11114477; doi:10.1093/gbe/evae098)
Supplement: evae098_Supplementary_Data [file evae098_supplementary_data.zip › Supplementary_materials.zip!/selected_miRNAs/DaRe_selected_miRNas/DaRe_NC_007128.7_30735.pdf]

Provisional ID	:	NC_007128.7_30735
Score total	:	114.4
Score for star read(s)	:	3.9
Score for read counts	:	107.7
Score for mfe	:	1.8
Score for randfold	:	1.6
Score for cons. seed	:	-0.6
Total read count	:	223
Mature read count	:	177
Loop read count	:	0
Star read count	:	46

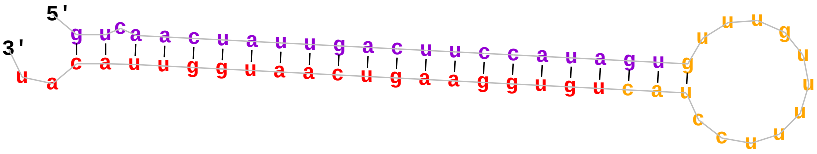

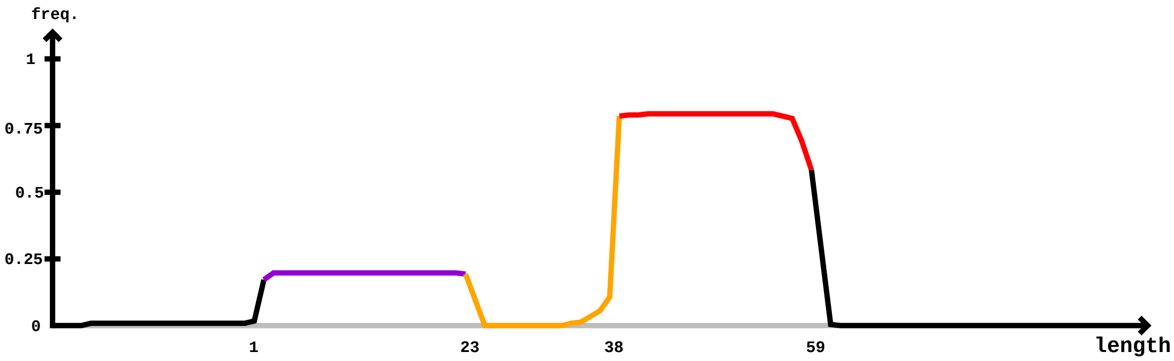

Star **Mature**

```
5'- augcauuuuugaugaaaggugucaacuauugacuuccauaguguuuguuuuuccuacuguggaagucaaugguuacaucauucuucaaaauaucuacuuuugggguuuugggu  -3'  obs
    augcauuuuugaugaaaggugucaacuauugacuuccauaguguuuguuuuuccuacuguggaagucaaugguuacaucauucuucaaaauaucuacuuuugggguuuugggu        exp
    ......((((((.(((.(((((.(((((((((((((((((((((..........)))))))))))))))))))))))))).))).)))))).(((((....)))))....... reads  mm  sample
    ....................gucaacuauugacuuccauagu....................................................................... 2  0  Gu2
    ....................................................ccuacuguggaagucaaugguA....................................... 2  1  Gu2
    ........................................................cuguggaGgucaaugguuac..................................... 1  1  Gu2
    ........................................................cuguggaGgucaaugguuaca.................................... 2  1  Gu2
    .........................................................uguggaGgucaaugguuac..................................... 1  1  Gu2
    .........................................................uguggaagucaaugguuacU.................................... 1  1  Gu2
    .........................................................uguggaGgucaaugguuaca.................................... 2  1  Gu2
    .........................................................uguggaagucaaugguuaca.................................... 2  0  Gu2
    .........................................................uguggaagucaaugguuacau................................... 11  0  Gu2
    .........................................................uCuggaagucaaugguuacau................................... 1  1  Gu2
    .........................................................uguggaagucaaugguuacauU.................................. 2  1  Gu2
    .........................................................uguggaagucaaugguuacauA.................................. 1  1  Gu2
    ..........................................................guggaagucaaugguuacaGc.................................. 1  1  Gu2

    ...................ugucaacuauugacuuccauagu....................................................................... 2  0  Ov3
    ....................gucaacuauugacuuccauagu....................................................................... 14  0  Ov3
    .....................ucaacuauugacuuccauagu....................................................................... 2  0  Ov3
    .......................................................aUuguggaagucaaugguuaca.................................... 1  1  Ov3
    ........................................................cuguggaaguGaaugguua...................................... 1  1  Ov3
    .........................................................uguggaGgucaaugguuac..................................... 1  1  Ov3
    .........................................................uguggaagucaaugguuaca.................................... 1  0  Ov3
    .........................................................uguggaagucaaugguuacau................................... 26  0  Ov3
    .........................................................uguggaagucaCugguuacau................................... 1  1  Ov3
    .........................................................uguggaagucaaugguuacauU.................................. 3  1  Ov3
    .........................................................uguggaagucaaugguuacauA.................................. 1  1  Ov3

    ....................gucaacuauugacuuccauagu....................................................................... 1  0  Gu1
    .....................ucaacuauugacuuccauagu....................................................................... 1  0  Gu1
    .....................................................cuacuUuggaagucaaugguuaca.................................... 1  1  Gu1
    ......................................................uacuguggaGgucaaugguuac..................................... 2  1  Gu1
    .......................................................acuguggaGgucaaugguuac..................................... 1  1  Gu1
    .......................................................acuguggaagucaGugguuac..................................... 1  1  Gu1
    ........................................................cuguggaGgucaaugguuaca.................................... 1  1  Gu1
```

```
                    Star                                Mature
augcauuuuugaugaaaggugucaacuauugacuuccauaguguuuguuuuuccuacuguggaagucaaugguuacaucauucuucaaaauaucuacuuuuggguuuugggu
........................................................cuguggaagucaaugguuaca...................................    2    0    Gu1
........................................................cuguggaagucaaugguuacU...................................    1    1    Gu1
.........................................................uguggaagucaaugguuac....................................    1    0    Gu1
.........................................................uguggaagucaaugguuGc....................................    1    1    Gu1
.........................................................uguggaGgucaaugguuac....................................    7    1    Gu1
.........................................................uguggaGgucaaugguuaca...................................    1    1    Gu1
.........................................................uguggaagucaaugguuaca...................................    1    0    Gu1
.........................................................uguggaagucaauUguuaca...................................    1    1    Gu1
.........................................................uguggaagucaaugguuacCu..................................    2    1    Gu1
.........................................................uguggaagucaaugguuacau..................................    24   0    Gu1
.........................................................uguggaGgucaaugguuacau..................................    1    1    Gu1
.........................................................uguggaagucaaugguuacauU.................................    4    1    Gu1
............................................................ggaagucaaugguuacaGca................................    1    1    Gu1

....................gucaacuauugacuuccauagu......................................................................    1    0    Gu3
.....................ucaacuauugacuuccauag.......................................................................    1    0    Gu3
......................................................uacuguggaGgucaaugguuac....................................    1    1    Gu3
......................................................uacugugUaagucaaugguuac....................................    1    1    Gu3
.......................................................acuguggaagucaGugguuac....................................    1    1    Gu3
........................................................cuguggaagucaaugguuaca...................................    1    0    Gu3
.........................................................uguggaGgucaaugguuaca...................................    2    1    Gu3
.........................................................uguggaagucaaugAuuacau..................................    1    1    Gu3
.........................................................uguggaagucaaugguuacCu..................................    1    1    Gu3
.........................................................uguggaagucaaugguuacau..................................    12   0    Gu3

..gcauuuuugaugaaaggug...........................................................................................    1    0    Ov1
....................gucaacuauugacuuccauagu......................................................................    9    0    Ov1
.....................ucaacuauugacuuccauagu......................................................................    2    0    Ov1
........................................................cuguggaagucaaugguuacau..................................    1    0    Ov1
.........................................................uguggaGgucaaugguuaca...................................    1    1    Ov1
.........................................................uguggaagucaaugguuacGu..................................    1    1    Ov1
.........................................................uguggaagucaaugguuacau..................................    11   0    Ov1
.........................................................uguggaagucaaugguuacauU.................................    1    1    Ov1

..gcauuuuugaugaaaggug...........................................................................................    1    0    Ov2
....................gucaacuauugacuuccauagu......................................................................    9    0    Ov2
.....................ucaacuauugacuuccauagu......................................................................    2    0    Ov2
......................................................uacuguggaGgucaaugguua.....................................    1    1    Ov2
.......................................................aUuguggaagucaaugguuac....................................    1    1    Ov2
........................................................cugugAaagucaaugguuaca...................................    1    1    Ov2
........................................................cuguggaagucaaugguuacau..................................    1    0    Ov2
.........................................................uguggaaguGaaugguuaca...................................    1    1    Ov2
.........................................................uguggaGgucaaugguuaca...................................    2    1    Ov2
.........................................................uguggaagucaaugguuacau..................................    28   0    Ov2
```
